# Towards MRI Study of Biointegration of Carbon-Carbon Composites with Ca-P Coatings

**DOI:** 10.3390/nano15070492

**Published:** 2025-03-26

**Authors:** Victoria V. Zherdeva, Petr E. Zaitsev, Andrei S. Skriabin, Alexey V. Shakurov, Vladimir R. Vesnin, Elizaveta S. Skriabina, Petr A. Tsygankov, Irina K. Sviridova, Natalia S. Sergeeva, Valentina A. Kirsanova, Suraya A. Akhmedova, Natalya B. Serejnikova

**Affiliations:** 1 Bach Institute of Biochemistry, Research Center of Biotechnology of the Russian Academy of Sciences, Moscow 119071, Russia; pez2000@yandex.ru; 2 Department of Power Engineering, Bauman Moscow State Technical University, Moscow 105005, Russia; terra107@yandex.ru (A.S.S.); shakurovalexey@gmail.com (A.V.S.); vesnin.volodya@gmail.com (V.R.V.); elzabra@yandex.ru (E.S.S.); 3 School of Physics, Industrial University of Santander, Bucaramanga 680002, Colombia; piotrtsy@mail.ru; 4 P.A. Herzen Moscow Research Oncology Institute, Branch of FSBI “National Medical Research Radiological Centre”, Ministry of Health of the Russian Federation, Moscow 125284, Russia; i.k.sviridova@yandex.ru (I.K.S.); prognoz.01@mail.ru (N.S.S.); kirik-57@mail.ru (V.A.K.); tagieva58@mail.ru (S.A.A.); 5 Institute of Regenerative Medicine, I.M. Sechenov First Moscow State Medical University, Moscow 119991, Russia; natalia.serj@yandex.ru

**Keywords:** carbon-carbon medical composites, hydroxyapatite coatings, electrophoretic deposition, detonation spraying, radiology studies, MRI, fibrous capsule, tissue response

## Abstract

The development of specific MRI criteria to monitor the implantation process may provide valuable information of individual tissue response. Using MRI and histological methods, the biointegration of carbon-carbon (C-C) composites into the subcutaneous tissues of BDF1 mice and their biocompatibility were investigated. The study focused on autopsy specimens containing C-C composite implants, both uncoated and coated with synthetic hydroxyapatite (Ca-P) via electrodeposition or detonation techniques, assessed at 6 and 12 weeks post-implantation. The results revealed that the radiological characteristics of the connective tissue capsule surrounding the implants allowed for the differentiation between loose and dense connective tissues. Fat-suppressed T1-weighted MRI scans showed that the volume of both loose and dense connective tissue in the capsule increased proportionally at 6 and 12 weeks, with distinct ratios observed between the coated and uncoated specimens. The proposed MRI criteria provided a strategy for evaluating the density and homogeneity of the connective tissue capsule. This approach could be valuable for further non-invasive in vivo studies on implant biointegration.

## 1. Introduction

Predominantly, strong and non-toxic metal structures (such as titanium, magnesium, or cobalt alloys) are applied for different surgical interventions into bone tissue and prosthetics of bone defects [1,2]. The implementation into clinical practice of various types of carbon materials, including carbon-carbon composites [3], is also considered a safe and non-toxic alternative for bone tissue implants and for healing of oncology diseases [4]. The applying of carbon structures allows a reduction in the specific weight of implants without a significantly deteriorating their exploitative characteristics that may be considered as an advantage of such materials.

Additionally, it can be noted that the applying of modern technological operations allows for the production of high-strength porous framework structures from carbon materials [5]. Some features of the morphology dictate interest in such implants, e.g., the porous structure could stimulate the cell proliferation on both the surface and deep into the implant [6] that may enhance the efficiency of biointegration.

Deposition of a number of bioceramic coatings (metal oxides, bioglasses, calcium phosphates Ca-P) may be recommended as an additional method of the integration enhancing [7]. As an option of deposited Ca-P compounds, tricalcium phosphates [8] and synthetic hydroxyapatite Ca-P [9] are considered currently as prospective materials for preparing bioactive layers on implants and stimulation of integration with no adverse reactions (prolonged inflammation, implant destruction, bacterial contamination, and tissue lyses) due to their high biocompatibility [10,11,12,13]. In the body, Ca-P crystals are dissolved under the interaction with the body fluids and actively metabolized to calcium and phosphorus ions, which subsequently may regenerate a novel bone tissue [14]. Porous carbon-carbon C-C composite substrates are folded by carbon fibers with amorphous carbon binding which provides a developed surface structure which may be covered with Ca-P layers [15] without toxic compounds.

Currently, detonation spraying [16] and electrophoretic deposition [17] are under consideration among the promising deposition techniques for such coatings. Detonation spraying of dispersed (with an average size of ≤50 μm) non- stoichiometric Ca-P particles allows us to prepare thick (up to 100 μm) low-porosity coatings of biocompatible ceramics based on a composition of Ca-P and tricalcium phosphate. As found for the electrophoretic technique and following heat post-treatment (annealing at 400 °C for 1 h), the preparing of calcium phosphate coatings did not lead to significant changes in the phase-chemical composition of the feedstock Ca-P due to a low heat loads during the process. A possibility to form coating in inner pores of the composite structures could be considered as a one of advantages of electrophoretic deposition, but adhesive strength of such coatings is less compared to detonation spraying.

The visual imaging capabilities of implant materials, including minimal artifact levels, support their high potential for clinical MRI translation. Carbon-carbon (C-C) implants meet these criteria as well [16,17], demonstrating biocompatibility and the absence of side reaction, as shown through micro-CT and micro-MRI studies [18,19].

MRI has previously been employed to assess the characteristics of implants. For example, the efficiency of cell colonization on implants was examined using iron oxide and superparamagnetic nanoparticles [19,20,21]. Additionally, contrast-enhanced MRI has been used to monitor the biocompatibility of implanted materials. Notably, Endorem-enhanced MRI was utilized to study the growth of bone marrow tissue on a polymer matrix [22].

The problem of reaction to an implant (so called a foreign body) after implantation is relevant for some groups of patients. Cellular events (macrophages and foreign body giant cells) with different chemical, physical, and morphological characteristics on synthetic surfaces of implants are considered to play a pivotal role in modulating tissue response reaction including fibrous capsule forming [23].

MRI has also been shown to detect fibrous tissue. Recent studies have demonstrated that quantitative mapping with MRI can evaluate the effectiveness of treatments for chronic liver fibrosis [24]. Additionally, myocardial fibrosis is detectable through gadolinium-enhanced MRI [25]. 3T MRI has been used to observe matrix remodeling and linearization of collagen in prostate tumors, a marker of active tumor growth, was detected using the EP-3533 probe [26].

These studies highlight that marker- or contrast-based MRI techniques can quantify fibrosis and assess matrix remodeling. To evaluate the tissue response to implantation, specifically the formation of the fibrous capsule, the development of appropriate MRI criteria is essential. Monitoring the formation of the recipient’s individual fibrous capsule in response to implantation could help prevent potential side effects.

Thus, the aim of work is to study coating deposition methods and their characteristics, establish MRI criteria for ex vivo assessing the integration of C-C implants into the body, and to compare the tissue responses between Ca-P-coated and uncoated samples.

## 2. Materials and Methods

### 2.1. Techniques of Coating Deposition and Their Characterization

As sphere-like micro-dispersed (with an average size of ≤60 nm for electrophoretic deposition and ≤50 μm for detonation spraying) feedstock particles, synthetic non-stoichiometric Ca-P (Biteka, Ltd., Odintsovo, Russia) was tested for the coating deposition on commercial C-C composites (NTM+, Ltd., Vsevolozhsk, Russia) in the form of cylinders with a diameter of ≈6 mm and a height of ≈3 mm. Using the detonation spraying technique [16], a Ca-P coating was deposited on the lateral composite surface at a frequency of 4 pulses per second. Electrophoretic deposition on the entire surface of the substrates was performed with [27] in an isopropyl alcohol environment (99.7%) at a constant voltage of 300 V and a deposition time of 1 min. Further, the samples were soaked in distilled water, dried, and annealed in an air muffle furnace at 400 °C for 1 h. The resulting samples were characterized by electron microscopy (Quattro microscope, Thermo Fisher Scientific, Waltham, MA, USA) at a magnification of ×400−3500. Visualization was performed in the secondary (SE) and backscattered (BSE) electron modes using the Everhart–Thornley and concentric back scattering detectors. Elemental analysis was performed using an energy-dispersive analysis system (EDAX Octane Elect plus, Shanghay, China). The roughness of the samples was studied using a Talysurf stylus profilometer (with a rounding radius of 2 μm of a diamond cone probe, measuring force of 70–100 mgf). The track length was selected as a sufficient size of the data collection zone (≈7 mm). A Gaussian filter was used to subtract the surface waviness.

An adhesive strength of the deposited coatings was measured with a LFM-50 electromechanical table machine (Walter and Bai, Löhningen, Switzerland) at a force of 0–50 N and a loading speed of 0–500 mm/min. The coatings were deposited on the flat composites with the diameter of 25 mm and the area of A_c_ ≈ 490 mm^2^. Total amount of the samples was 30 (3 groups; uncovered, detonation-sprayed, and electrophoresis-covered substrates; 10 samples per a group). The samples were fixed with a test assembly using epoxy resin (1 day drying under the room condition). An adhesive strength σ_a_ was calculated as σ_a_ = F_f_/A_c_. Here, F_f_ is the failure force [28].

### 2.2. Animal Implantation and Ex Vivo Sample Preparation

The sterilized (at 180° C for 90 min) control (uncoated) and experimental (Ca-P coated) samples were subcutaneously implanted in male laboratory BDF1 mice (Federal Medical-Biological Agency, Andreevka branch, Andreevka, Moscow region, Russia) weighed ≈ 18–20 g according to the procedure [27]. The study was conducted in accordance with the Declaration of Helsinki. The in vivo experiments on animals were authorized by the Commission for Bioethical Control of Content and Use of laboratory animals for scientific purposes (protocol #2—SI-00050, date of approval: 15 September 2023) of the FSBI “NMSC of Radiology” of the Ministry of Health of the Russian Federation.

The implantation caverns were formed on the back of the animals, and the surgical invasion was fulfilled under sterile conditions using the anesthesia with 0.1 mL ketamine/relanium mixture in an equal ratio. Each experimental group (uncovered, electrophoresis- and detonation-prepared composites) for each period contained 4 animals. At the 6th and 12th week of mice, euthanasia was subjected to CO_2_ camera (Techniplast, Buguggiate, Italy). The preparation of biopsy samples for histochemistry was carried out according to [27].

Six groups of animal (n = 4 per group) were analyzed using MRI slices of all grouped animals (N = 18–21).

The groups were the following:

- Autopsy of uncoated C-C composites at week 6 post-implantation;
- Autopsy of uncoated C-C composites at week 12;
- Autopsy of Ca-P coated (electrophoretic deposition) C-C composites at week 6 post- implantation;
- Autopsy of Ca-P coated (electrophoretic deposition) C-C composites at week 12 post- implantation;
- Autopsy of Ca-P coated (detonation spraying) C-C composites at week 6 post-implantation;
- Autopsy of Ca-P coated (detonation spraying) C-C composites at week 12 post-implantation.

Then, the harvested samples was paraffin-embedded, and histological sections with a thickness of 4 mm were stained by hematoxylin and eosin and studied with Leica DM 4000 B microscope (Leica Microsystems, Wetzlar, Germany) and Leica DFC 7000 camera under standard under brightfield and phase-contrast visualizations.

### 2.3. MRI Scanning of Ex Vivo Samples

The prepared ex vivo samples were scanned using the 1T M3 MRI tomography system (Aspect Imaging, Shoham, Israel). The samples embedded in 15 mL plastic tubes placed in an integrated radiofrequency body coil (with a length of 50 mm and a diameter of 38 mm). MRI images were acquired using T2w FSE (T2-weighted fast spin echo) and T1wGRE (T1-weighted gradient echo) pulse sequences with following parameters of TR/TE 4000/38.3, FOV 40 × 40, matrix 120 × 180, ETL 5, NEX 8, N = 15 and TR/TE = 60/2.4 ms, FA 30, NEX 20, FOV 40 × 40 mm, matrix 120 × 180, ETL 5, NEX 8, N = 15 correspondently. To suppress the fat tissue signal, an inversion prepulse of TI = 100 ms was used. Maximum intensity projection (MIP) images of a selected MRI slice stack (N = 18–21) were used to obtain segmented images for all experimental groups (n = 4 per group).

### 2.4. MIP Analysis for MR Images

ImageJ/Fiji 2.16.0, open software (NIH, Bethesda, MD, USA) with MRI processing toolbox was used for the image analysis. Built-in plugin maximum intensity projection (MIP) was applied for MR images analysis.

For images, processing maximum intensity projection (MIP) method with a pseudocolor intensity distribution, where 254 is the maximum intensity (Fire\Image J\Fiji) was used [28]. The values of the minimum and maximum intensity of the selected areas (N = 18–21 for every study) were analyzed using the linear separability method. The data array is linearly inseparable, so division by line was used where the boundary values are least in contact with the separation line [29].

Loose connective tissue (LCT) was determined in the range of 40–100 units of scale, while dense connective tissue (DCT) was represented by the color from 101 to 254 units, which visually corresponded to the histological tissue pattern of the ex vivo specimens.

### 2.5. Statistical Analysis

The differences in loose and dense connective tissue values were statistically significant (*p* ≤ 0.05) according to one-tailed non-parametric Mann–Whitney–Wilcoxon (MWW) *t*-test paired observation; data were analyzed using a one-tailed non-parametric (unpaired) MWW *t*-test, N = 18–21 data points per group. Multiple groups were compared using an ordinary one-way ANOVA test (Prism 8.4.3, GraphPad Software).

## 3. Results

### 3.1. Morphology of Composite Substrates and Deposited Coatings

**Figure 1:**
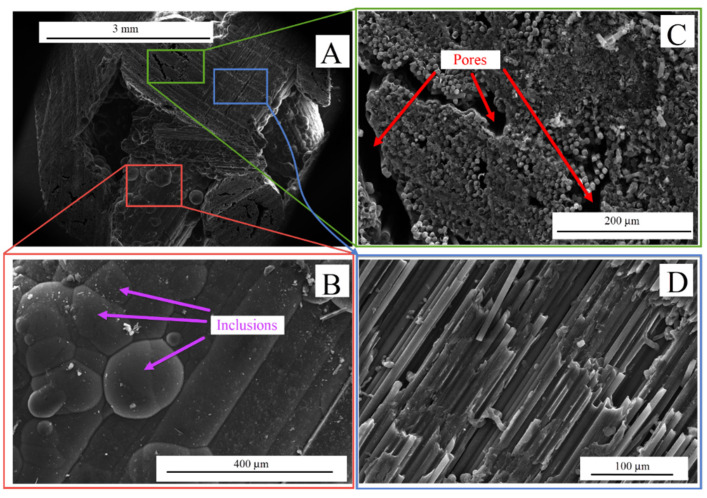
SEM images of C-C composite substrate captured at varied magnifications: (**A**) ×65; (**B**) ×800; (**C**) ×500; (**D**) ×1000.

The obtained electron images of the C-C composite substrate samples are shown in Figure 1 captured at different magnifications. The C-C composite structure (see Figure 1A) had a quite carvenous surface, which could be a basis for effective cell proliferation. An important feature of such structures was the presence of internal pores formed by the fibers. In addition, the implant surface was complicated by the presence of external rounded inclusions with a characteristic size of up to ≈200 μm (see Figure 1B). The substrate structure (see Figure 1C,D) was formed by the long (with a length of ≈1–3 mm) and thin (with a diameter of up to ≈8 μm) carbon fibers, which were laid into larger wires with a diameter of about 1 μm and connected in them using the amorphous carbon binder. These fibers were also intertwined into a single 3D framework. An important feature of the substrate morphology was the significant anisotropy of roughness along different directions. Along the fibers, the roughness was Ra = 1.56 ± 0.23 μm. The substrate roughness across the fibers was not accurately and reliably measured due to the significant influence of porosity.

**Figure 2:**
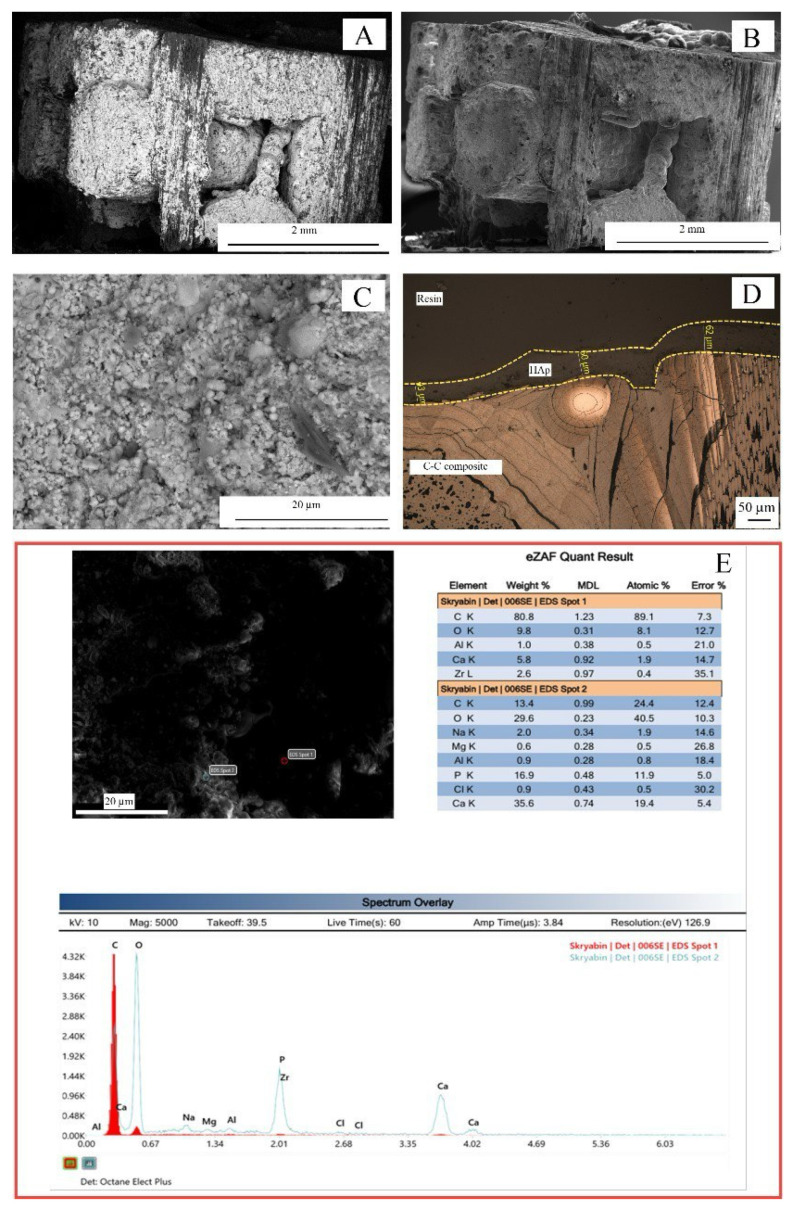
Ca-P coatings on C-C composite (detonation deposition): (**A**–**C**) SEM images; (**D**) their cross-section; (**E**) EDX data in indicated spots.

In the case of the detonation spraying (Figure 2), the coatings on the C-C composites demonstrated a tight fit of the deposited coating to their lateral surfaces. The coated area depended on the sample orientation relative to the high-speed dispersion flow with Ca-P particles. Visualization in the backscattered electron mode (Figure 2A) demonstrated a higher contrast of the coating on the carbon substrate compared to the secondary electron mode visualization (Figure 2B). It was found that the coating had been formed by particles that experienced plastic deformation during deceleration of the initial spherical particles with sizes of several microns (Figure 2C). Microscopic study of the cross-sections (Figure 2D) showed that the thickness of the deposited coating was ≈43–62 μm. The coating mainly was found on the outer surface of the implant without a penetrating deep into the internal pores. The procedure for preparing the cross-sections did not lead to noticeable fracturing and chipping of the coating. The energy dispersive analysis data (Figure 2E) showed that the elemental composition of the coating included elements typical for Ca-P bioceramics (Ca, P, and O), as well as the presence of C due to the influence of the substrate. The trace inclusions of other elements (Al, K, etc.) did not seem to be significant. The stoichiometric Ca/P ratio was Ca/P ≈ 1.55–1.63. The roughness along the coated fibers was Ra = 6.11 ± 2.33 μm as found.

**Figure 3:**
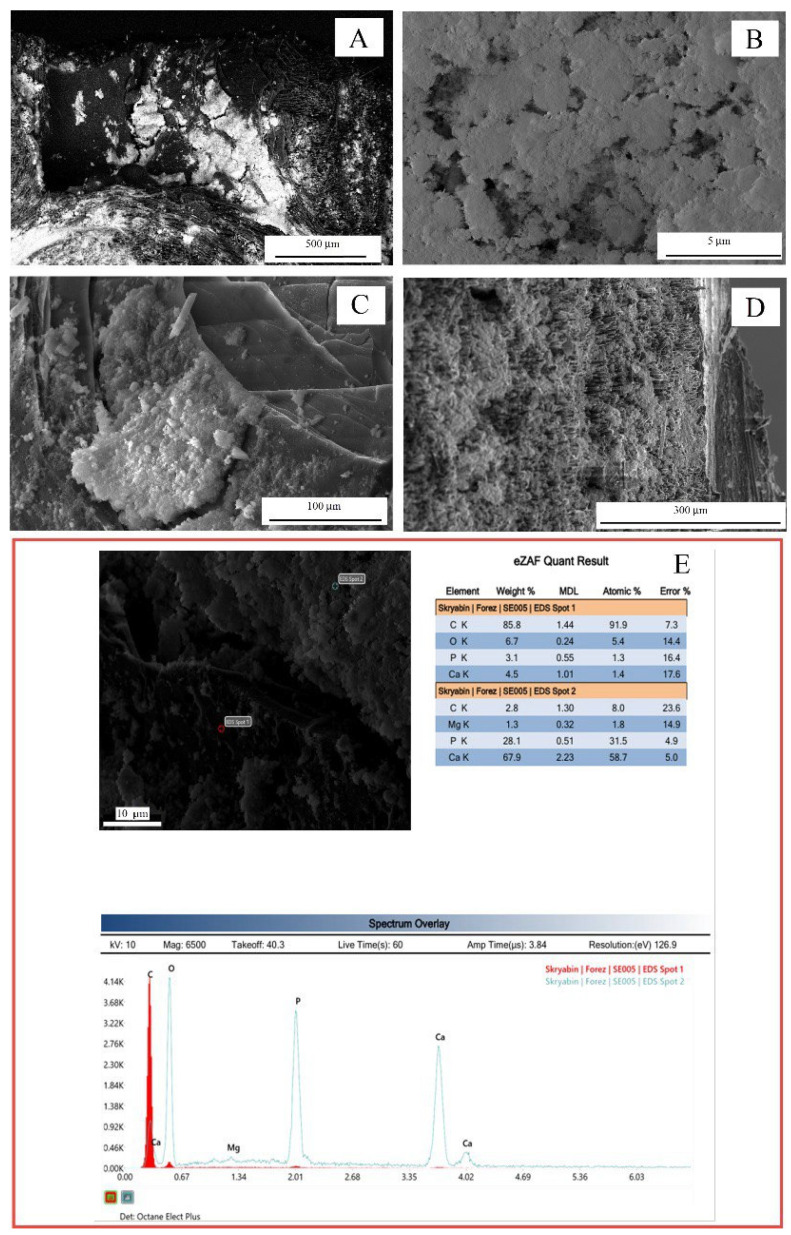
Ca-P coatings on C-C composite (electrophoretic deposition): (**A**–**D**) SEM images; (**E**) EDX data in indicated spots.

For the coatings obtained by electrophoretic deposition, the formation of flake-like Ca-P structures without a tight adhesion to the carbon substrate was observed (Figure 3A). The presence of numerous pores in the coating with numerous failures (Figure 3B) was found. The adhesion was not continuous (Figure 3C), unlike the coating obtained by detonation spraying when penetration of Ca-P into internal pores and cracks was visualized (Figure 3D). As in the previous case of detonation spraying, the same elements were detected in the coatings as presented in Figure 3E. However, the stoichiometric ratio Ca/P was different and was Ca/P ≈ 1.72–1.86. The coating roughness was close to the substrate roughness (Ra = 1.56 ± 0.23 μm) due to the rather weak adhesive strength of the coatings and the relatively random coating distribution over the substrate.

Summary, the most important findings from SEM images and EDX evidenced that detonation spraying led to deposition of a tight fitting coating, which covered an outer surface of the composite substrate due to the superficial impact of heterophase detonation flow with the dispersed particles. The coating had a compacted structure formed by plastically deformed HAp particles, which were accelerated in the detonation flow and then decelerated on the substrate. As a result, the coating with a relatively low porosity and a thickness of ≈43–62 μm were prepared. As a type of electrochemical deposition, the deposited coatings had a loose structure, which was prone to crumbling due to a low heat loads and in the absence of the interaction of the dispersed high speed particles with the substrates. We found an isolated coating deposition, which led to the presence of uncovered areas of the composites. The recorded Ca/P ratios were ≈1.55–1.63 (detonation spraying) and ≈1.72–1.86 (electrophoretic deposition), which were close to the reference range of Ca/P (≈1.61–1.76) [28]. The found small difference was not critical for the prospective of the deposition techniques and was determined by the deposition procedure which was focused on plasma-sprayed coatings in the standard.

Such structure features of the deposited coatings determined the values of their adhesive strength. As found, the σ_ad_ values for the detonation-sprayed coatings were 0.747 ± 0.419 MPa. The found σ_ad_ values were slightly lower than the other data on the HAp coatings on the carbon-forced composites [30].

In the case of electrophoresis-deposited coatings, the found adhesive strengths were approximately 10 times lower (no more than σ_ae_ = 0.083 ± 0.061 MPa). Here, we note the obtained σ_ae_ values could even exceed the real values due to the substrate influence during the tests. The adhesive strength of the uncovered composites was σ_as_ = 1.802 ± 0.776 MPa.

As found earlier [16], the detonation spraying led to a number of physical and chemical transformations of feedstock non-stoichometric HAp into stoichometric HAp and α- and β-tricalcium phosphates and amorphouse phosphates were detected in the prepared coatings too. In this case, the crystallinity index CI and the average crystallite size d were about 23% and 22.2 nm, respectively. Electrophoretic deposition and the following heat treatment did not change significantly the phase composition of coatings, which was closed to feedstock HAp (with CI ≈ 25% and d ≈ 16.5 nm) [27]. XRD data on the phase compositions were confirmed with Raman spectroscopy [16,27]. None of the cytotoxic compounds were detected for both techniques.

### 3.2. MIP Analysis

**Figure 4:**
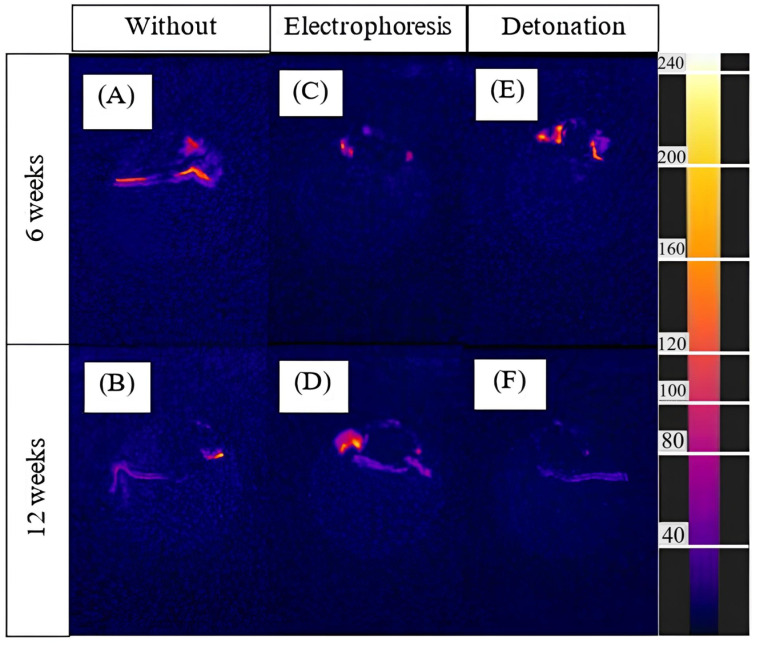
Pseudocolored mapping of T1w-TI MR-images (TI prepulse = 100 ms) of the C-C composites: (**A**) uncoated, 6 weeks; (**B**) uncoated, 12 weeks; (**C**) Ca-P coated (electrophoretic deposition), 6 weeks; (**D**) Ca-P coated (electrophoretic deposition), 12 weeks; (**E**) Ca-P coated (detonation spraying), 6 weeks; (**F**) Ca-P coated (detonation spraying), 12 weeks. Fire color filter (Image J/Fiji).

Figure 4 represents the examples of Fire images processing. Post-processing of T1w-TI scans showed the only connective tissue at every Fire-image while the signal from a fat was suppressed (Figure 4A–F). Figure 4A,B show partial capsule formation for the control samples. For samples prepared by electrophoretic deposition (Figure 4B,D) and detonation spraying (Figure 4E,F), dense connective tissue appears as a rich yellow-red spot. Post-processing of T1w-TI scans was applied for all samples routine processing.

The MIP projections of all MR scans were processed and loose (corresponded to the 0–100 a.u. at the Fire scale) and dense (corresponded to the 101–254 a.u. at the Fire scale) areas were summarized for every sample (N = 10–11). Dense connective tissue forms a characteristic circumferential capsule with electrophoresis spraying and distal growth with detonation spraying.

**Figure 5:**
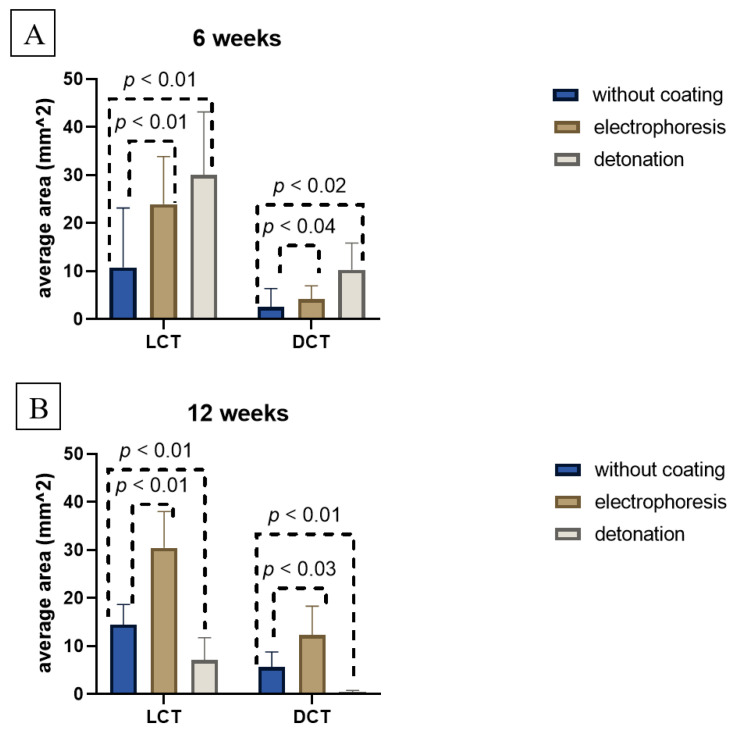
Averaged area of loose (LCT) and dense (DCT) connective tissue of fibrous capsule in autopsy samples taken at 6-week (**A**) and 12-week (**B**) post-implantation, with different coatings (uncoated, electrophoretic deposition, detonation spraying) (4 mice per group, number of all analyzed sections N = 18–21). *p* value < 0.05 is statistically significant for differentiation of loose and dense connective tissue in all samples.

Figure 5 demonstrates the summarizing value of loose and dense connective tissue. Figure 5A,B show the dominance of loose tissue over dense tissue for all samples at all observation periods: at 6 weeks (Figure 5A) and at 12 weeks (Figure 5B) of the post-implantation period, respectively. The tendency of fibrous capsules enlargement from uncoated to coated samples was observed (Figure 5).

#### 3.2.1. Uncoated C-C Composites

**Figure 6:**
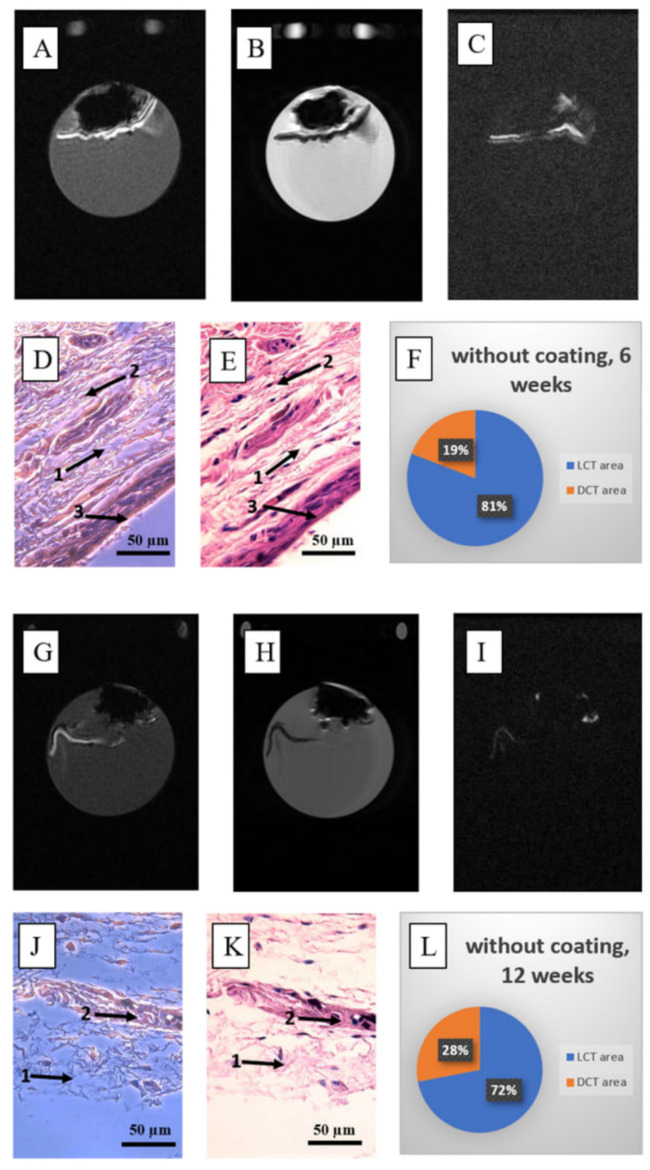
The features of the fibrous capsule for uncoated C-C composites (control) at 6 weeks (**A**–**F**) and 12 weeks (**G**–**K**) post-implantation are as follows: (**A**,**G**) T1-weighted (T1w) MR image of the central section; (**B**,**H**) T2-weighted (T2w) MR image of the central section; (**C**,**I**) T1w-TI MR image of the central section; (**D**,**E**,**J**,**K**) hematoxylin and eosin staining of the post-implantation autopsy sample (magnification ×400), where 1 = loose tissue, 2 = dense tissue with fragments of C-C composites, 3 = site of tissue contacts with the implant; (**F**,**L**) diagram representing the ratio of loose connective tissue (LCT) to dense connective tissue (DCT) for the entire autopsy sample (number of slices N = 18–21).

T1wGRE (Figure 6A,G), T2w FSE (Figure 6B,H), and TI prepulse = 100 ms (Figure 6C,I) MRI demonstrated a partial capsule formation around the uncoated scaffold in different ex vivo samples obtained at 6 and 12 weeks, respectively. This was confirmed by histological studies demonstrated as parts of a loose connective tissue and as parts of a dense connective tissue (Figure 6D,E,J,K and Figures S1–S3). For the uncoated samples, the percentage of dense tissue corresponded to 10% at 6 weeks’ observation (Figure 6F) and 19% at 12 weeks’ observation from a whole volume (Figure 6L).

#### 3.2.2. Ca-P Electrophoretic Deposition

**Figure 7:**
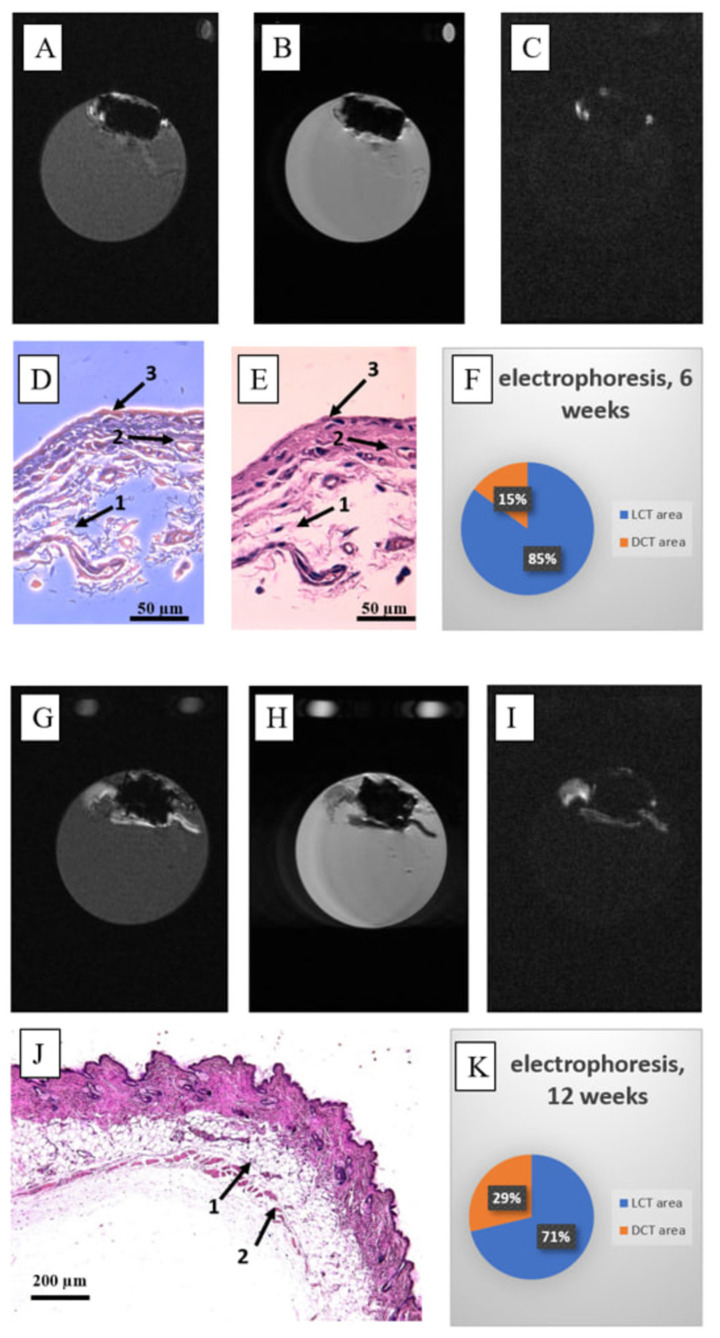
The features of the fibrous capsule for C-C composites coated with Ca-P via electrophoretic deposition at 6 weeks (**A**–**F**) and 12 weeks (**G**–**K**) post-implantation are as follows: (**A**,**G**) T1-weighted (T1w) MR image of the central section; (**B**,**H**) T2-weighted (T2w) MR image of the central section; (**C**,**I**) T1w-TI MR image of the central section; (**D**,**E**,**J**) hematoxylin and eosin staining of the post-implantation autopsy sample (magnification ×400), where 1 = loose tissue, 2 = dense tissue, 3 = site of tissue contacts with the implant; (**F**,**K**) diagram representing the ratio of loose connective tissue (LCT) to dense connective tissue (DCT) for the entire autopsy sample (number of slices N = 18–21).

For the samples coated with Ca-P by electrophoretic deposition, a whole capsule was visualized on MR scans along the entire perimeter of the scaffold (Figure 7). High contrasting of loose and dense tissue were clearly visible on the stained slices (Figure 7D,E,J and Figures S4 and S5). The percentage of dense tissue increased (Figure 7K) compared to the uncoated samples (Figure 6L) and the absolute volume of the capsule was increased as well in comparing with the uncoated samples (Figure 2).

#### 3.2.3. Ca-P Detonation Spraying

**Figure 8:**
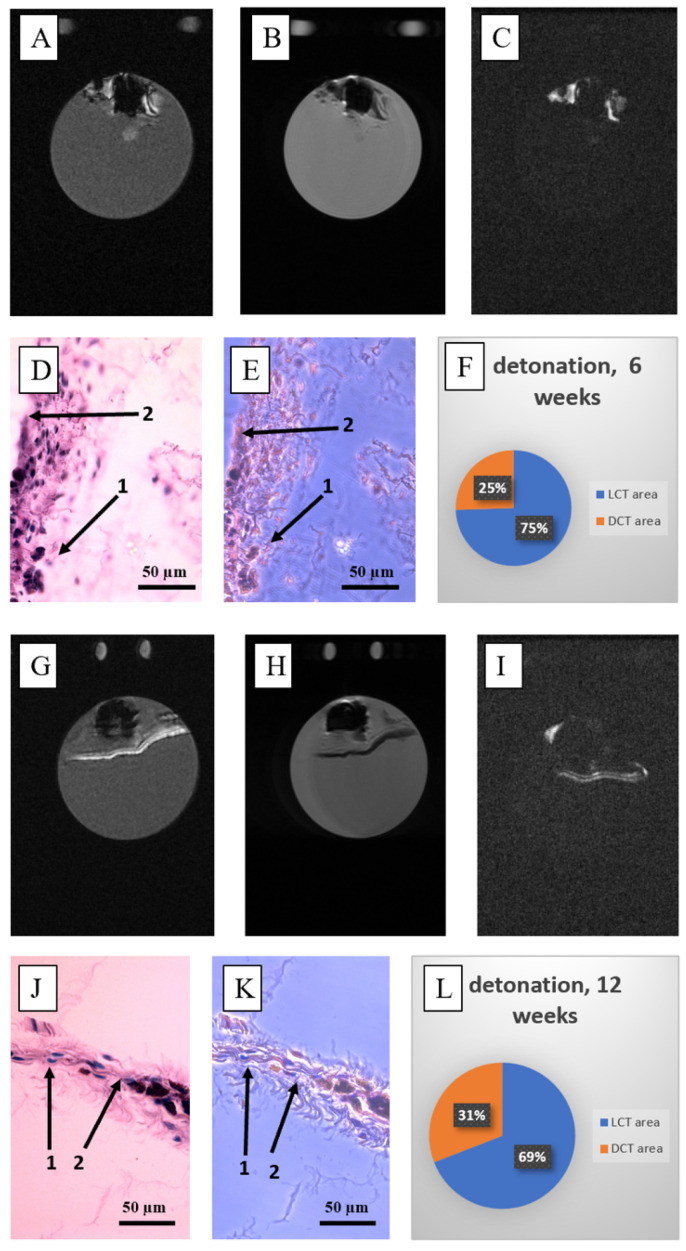
The features of the fibrous capsule surrounding C-C composites coated with Ca-P via detonation spraying at 6 weeks (**A**–**F**) and 12 weeks (**G**–**K**) post-implantation are as follows: (**A**,**G**) T1-weighted (T1w) MR image of the central section; (**B**,**H**) T2-weighted (T2w) MR image of the central section; (**C**,**I**) T1w-TI MR image of the central section; (**D**,**E**,**J**,**K**) hematoxylin and eosin staining of the post-implantation autopsy sample (magnification ×400), where 1 = loose tissue, 2 = dense tissue with fragments of C-C composites; (**F**,**L**) diagram illustrating the ratio of loose connective tissue (LCT) and dense connective tissue (DCT) for the entire autopsy sample (namber of slices N = 18–21).

For detonation spraying, the peri-implant tissues formed extensive distal areas at the edges of the scaffold, where the main coating was concentrated (Figure 8A–C,G–I). Histological analysis showed a poorly developed capsule in areas where hydroxyapatite was deficient (Figure 8D,E,J,K), and these regions exhibited the highest percentage of dense connective tissue (≈31–32% vol.) at both 6 and 12 weeks post-implantation (Figure 8F,L). The capsule volume was significantly increased compared to the uncoated and electrophoretic samples (Figure 2). Fragments of C-C implants were detected more frequently in these samples than in the uncoated ones. Additionally, the capsules was enriched with lymphocyte and macrophage infiltration (Figures S6 and S7). At 12 weeks, some capsules showed mechanical lesions, affecting their measurements.

When compared to electrophoretic deposition, an increase in dense tissue at the 12-week post-implantation mark was observed, but the loose to dense connective tissue ratio for the detonation-sprayed coatings remained stable at approximately 30% vol.

## 4. Discussion

As is well known, the implant–host integration process consists of acute and chronic reactions [31]. The acute phase is characterized by macrophage and lymphocyte infiltration and, as a consequence, the development of the inflammatory process. Then, the implant (foreign body) is surrounded by fibrous tissue, and the process finished with the formation of the fibrous capsule around the implant [31]. Typically, these tissue effects are studied on biopsy or autopsy samples using pathomorphological analysis. In this regard, the application of non-invasive methods has recently received more attention due to the emphasis to personalized approaches in medicine [32]. The ability to image ex vivo samples at high resolution was demonstrated in our previous study [32].

In the present study, 1T MRI was used to visualize tissue effects in autopsy samples containing carbon-carbon composites. Three groups of samples were compared: uncoated samples and coated by detonation spraying or electrophoretic deposition with synthetic Ca-P composition. The samples were dissected at 6 and 12 weeks post-implantation in BDF1 mice. As a result of the study, MR criteria for the ratio of loose and dense connective tissue (LCT to DCT) in the fibrous capsule around implants ex vivo were proposed. The samples buffered in formalin were contrasted in T1w with fat suppression (100 ms TI-T1w pulses). The presence of loose and dense connective tissue around the ex vivo samples was confirmed by pathomorphological studies [27]. MRI visualized whole tissue effects while histology allowed detecting separate areas of autopsy samples. The dense connective tissue demonstrated high hyperintensity and integral signal density in comparing with loose tissues in TI-T1w mode. The contrast was enhanced by image processing with the Fire pseudocolor mode in Image Fiji software (Figure 4).

The dense connective tissue increased proportionally from 10 to 19% in volume in uncoated samples over 6 and 12 weeks post-implantation. Electrophoretic deposition with non-continuous adhesion characteristics of Ca-P coatings resulted in a slight compaction of the capsule and the volume of dense connective tissues was varied from 18 to 23%. As was found for detonation Ca-P spraying, the LCT/DCT ratio was shifted to the dense tissue (up to 32% of a whole fibrous volume) for all studied samples over 6 weeks of observation. Fibrous capsule was found to increase 2–2.5 at 6 weeks post-implantation for all coated samples compared with uncoated. According to histological studies, an increased level of macrophages and lymphocytes infiltration was noted. The capsules of uncoated samples were thin and formed versus capsules of coated samples shown to be thick and enriched with dense connective tissue and high level of lympho- and macrophagal infiltration. The loose/connective tissue ratio for the detonation-sprayed coatings aroused to 30% at 6 weeks of post-implantation. Revision of histological autopsy samples at 12 weeks demonstrated some quantity of the implanted materials incorporated in the giant macrophages. This may probably indicate the process of a digestion of large fragments of implanted materials.

Differences in tissue reactions in response to the sample integration require analysis of the coating procedure. Ca-P may presumably influence tissue responses through modulation of immune responses by particle size and Ca-P, as discussed previously [33]. It is obvious, that variable parameters of the roughness along the coated fibers of Ra = 6.11 ± 2.33 μm and the adhesive strength of σ_ad_ = 0.747 ± 0.419 MPa for detonation-sprayed coatings; and Ra = 1.56 ± 0.23 μm (the last one is closed to uncoated substrate roughness) and σ_ae_ = 0.083 ± 0.061 MPa for electrophoretic-deposited coatings may impact on recognition by immune cells. It may recruit different cell events, for example, endocytosis (particles < 0.5 μm in size) and phagocytosis (particles larger than 0.5 μm) [33]. Most probably, the endocytosis prevaleged in utilizing the Ca-P small size particles deposited by electrophoresis, while the large particles formed as a result of the detonation spraying were utilized mostly by phagocytosis and led to the formation of giant phagocytes.

As shown earlier, not only the size, but also the shape of nanoparticles is rather important for recognition by immune cells. It is shown that rounded particles are preferably recognized by cells [33]. Thus, the balance of Ca-P and size of nanocoatings determines the active cellular microenvironment and modulate the response. The best biocompatibility by all criteria was demonstrated by implants that remained in the body for a long time and were resorbed without the formation of extensive fibrous scars. This was facilitated by the rough surface of the implants, which increased the cell adhesion. It has been reported that the optimal intermediate roughness ratio r for the effective cell growth was r = 2 [34].

At the same time, higher coating density, its adhesive strength, and thickness up to 100 μm of detonation spraying caused more extensive foreign body reaction in comparing to electrophoretic deposition as it was revealed by MRI results and histological study. When analyzing the MRI scans, the effect of the prevalence of the dense connective tissue was noticed at the edge of the detonation spraying implant (Figure 8). Presumably, it was due to the coating procedure that the detonation spraying did not cover all sample surfaces but preferably was found at the curved surface of the C-C cylindrical samples, which contributed less to the biointegration of the implant [35]. The chronic fibrous capsule will likely prevent biointegration of therapeutic function of implants on a long-time scale [32]. It is well known of acute and chronic stages of a fibrous capsule mechanisms forming, including different steps and cell pools engagement, but less is known about “feedback’’ from implants to the tissue and immune microenviroment.

The preliminary toxicity in vitro and in vivo assessment is an important step of the implanted materials study. There are no certain standards to identify toxicity. Factors such as the local legislation, the applicable standards, and the intended location of the device influence the specific tests that need to be performed.

The use and obligatory testing of medical products for implantation is regulated by international and local legislation. New materials are subjected to laboratory tests. Tests may differ depending on the task. Tests on biocompatibility are usually based on in vitro and in vivo including mechanical, chemical, and biological properties studies [36].

Tests for cytotoxicity, genotoxicity, and blood tests when evaluating such new materials are very common [37,38,39].

Blood tests in this study were not carried out, but earlier studies of one of the materials with electrophoretic spraying indicate the absence of cell toxicity and genotoxicity [27].

The main objective of this study is to use the radiological method of low-field 1T MRI to evaluate connective tissue as a marker of fibrous capsule formation. Earlier the high-field MR imaging was used to solve similar problems and study fibrosis in adipose tissue ex vivo [39]. Thus, the studies of the human biopsy samples (with a volume of about 1 cm^3^) taken from patients with morbid obesity were carried out at the 4.0 T MR tomograph (3D-MRI Bruker, Billerica, MA, USA). The results of this MR mapping of fibrosis in the subcutaneous adipose tissues correlated with the histology results with a high degree of reliability [40]. As shown earlier, MRI demonstrated a high sensitivity in combination with specific contrast agents. MRI was applied to determine the degree of collagen linearization in prostate tumors as a marker of active tumor growth with the contrast agent EP-3533 [41].

In the present study, we obtained data on the density of fibrous tissues around the implants using maximal intensity projection (MIP) to estimate the intensity of MRI slices. The MR structure of a collagen was enhanced by T1w+TI modes. MIP calculations were also effective for identifying the maximum possible values for special effects; for example, these approaches were used in cell analysis, e.g., to study the location of cells and neuronal growth [21,22]. This approach is highly effective to demonstrate the effects of stacking MRI and optical slices [42].

To design high sensitive MRI methods, the initially ex vivo study is required. MR criteria for assessment of the loose and dense connective tissues of the fibrous capsules were justified for three types of C-C composites. The proposed MR criteria for determining the efficiency of biointegration, based on the LCT per DCT ratio, demonstrated the potential for their application in MR histological validation. The method objectively assessed the ex vivo several test samples implanted in soft tissues. In the future, it will be necessary to examine in vivo samples located in bone tissues using the same method and compare the presented results with the bone implantation.

Combining MRI to other modalities may provide highly sensitive and informative real-time visualization of scaffolds’ biointegration, cell colonization, and tissue response assessment simultaneously. For example, combining MRI to optical imaging with MR contrast agent gadobutrol leads to the significant improvement of the fluorescence visualization of cells marked with a genetically encoded probe as a diffusion coefficient estimation in tissue provided by different compositions of optical clearing agents [43]. To demonstrate imaging depth and imaging contrast, the improvement of a number of osmotic solutions may be used, like glycerol, glucose, and albumin [44]. At the same time, they may help to track drug delivery when implanting tissue-engineered structures with controlled prolonged drug delivery supported by MRI and optical imaging.

It should be noted that MRI and MIP analysis for the differentiating of two types of connective tissue requires further study on extended animal cohorts. The method is suggested in combination with histology to confirm the dense and loose connective tissue in the sample, but allowing the calculation of the exact area and density of tissue with known delineations obtained by histological methods. This approach is similar to the 3D modeling of tissues previously proposed for fluorescence microscopy results mapping. The optical clearing special approach was used in this work to enhance the contrast of the tissue [45].

Here in our work, the formalin fixation improves the contrast of connective tissue in 1T MRI [27]. The main results of this work are the combination of MRI and the approach to MIP, which allows us to differentiate loose and dense connective tissue of the fibrous capsule ex vivo, which is formed around a foreign body (implanted material). For a similar assessment of the ratio of loose and dense connective tissue, but already in vivo using the same MRI 1T of a low field, the highly sensitive approaches using contrasting agents should be developed.

## 5. Conclusions

MR criteria for the histological validation of the fibrous capsule of carbon-carbon (C-C) composites ex vivo were proposed. Fat-suppressed T1-weighted MRI revealed that the volume of loose and dense tissue in the capsule surrounding the implants increased proportionally at 6 and 12 weeks, with distinct ratios observed for coated versus uncoated samples. The ratio of loose to dense connective tissue forming the capsule serves as an indirect indicator of the biocompatibility and biointegration of the implanted scaffolds.

Furthermore, the shape and surface roughness of calcium–phosphate (Ca-P) coatings significantly influenced the thickness and ratio of loose to dense connective tissue in the fibrous capsule at 6 and 12 weeks post-implantation.

Notably, highly rough surfaces and thick deposition sprays on coated C-C samples led to a 2.5- to 3-fold increase in the volume of the fibrous capsule compared to uncoated samples. Electrophoresis was characterized by the distribution of a fibrous capsule along the perimeter of the frame and it was shown that it has the best functions of integration, due to the smaller coefficients of roughness, despite the smaller adhesive strength.

The proposed MR criteria facilitated the development of an evaluation strategy to assess the density and homogeneity of the connective tissue capsule. While the histology allows detecting separate areas of autopsy samples, MRI enables visualizing whole tissue effects. Our preliminary results gave the idea to use such an approach in differentiating between fibrous tissue types but ex vivo. Following experiments in vivo would give more reasons for capturing subtle biointegration difference, comparison, and subsequent validation.

The integration of MRI with other methods could ensure the real-time visualization of the scaffold biointegration, cell colonization, and the smart management of tissue response.

## Data Availability

The original contributions presented in this study are included in the article/Supplementary Material. Further inquiries can be directed to the corresponding author(s).

## Supplementary Materials

The following supporting information can be downloaded at: https://www.mdpi.com/article/10.3390/nano15070492/s1, Figure S1. Autopsy of uncoated C-C composites at week 6 post-implantation. (a) Standard light microscopy. Increase 50×. (b) standard light microscopy. magnification 200×. (c) standard light microscopy. 400×. (d) phase-contract microscopy. 400×. H& E statining. Figure S2. Autopsy of uncoated C-C composites at week 6 post-implantation with increased lymphocyte and macrophage infiltration. (a) Standard light microscopy. Increase 50×. (b) standard light microscopy. magnification 200×. (c) standard light microscopy. 400×. Figure S3. Aautopsy of uncoated C-C composites at week 12. (a) Standard light microscopy. Increase 50×. (b) standard light microscopy. magnification 200x. (c) standard light microscopy. 400×. (d) phase-contract microscopy. 400×. H& E statining. Figure S4. Autopsy of Ca-P coated (electrophoretic deposition) C-C composites at week 6 post-implantation. (a) Standard light microscopy. Increase 50×. (b) standard light microscopy. magnification 200×. (c) standard light microscopy. 400×. (d) phase-contract microscopy. 400×. H& E statining. Figure S5. Autopsy of Ca-P coated (electrophoretic deposition) C-C composites at week 12 post-implantation. (a) Standard light microscopy. Increase 50×. H& E statining. Fgure S6. Autopsy of Ca-P coated (detonation spraying) C-C composites at week 6 post-implantation. (a) Standard light microscopy. Increase 50×. (b) standard light microscopy. magnification 200×. (c) standard light microscopy. 400×. (d) phase-contract microscopy. 400×. H& E statining. Figure S7. Autopsy of Ca-P coated (detonation spraying) C-C composites at week 12 post-implantation. (a) Standard light microscopy. Increase 50×. (b) standard light microscopy. magnification 200×. (c) standard light microscopy. 400×. (d) phase-contract microscopy. 400×. H& E statining.
