# Supplementary material for: Towards MRI Study of Biointegration of Carbon-Carbon Composites with Ca-P Coatings
Source: Nanomaterials (Basel). 2025 Mar 26;15(7):492. doi: 10.3390/nano15070492 (PMC11990118; doi:10.3390/nano15070492)
Supplement: Supplementary file 1 [file nanomaterials-15-00492-s001.zip › nanomaterials-3490242-supplementary.pdf]

a)

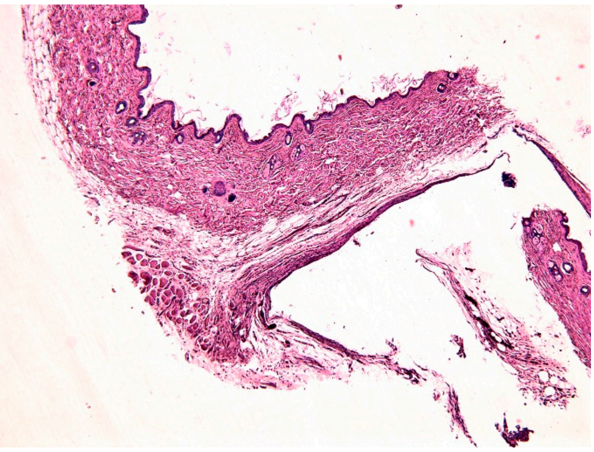

b)

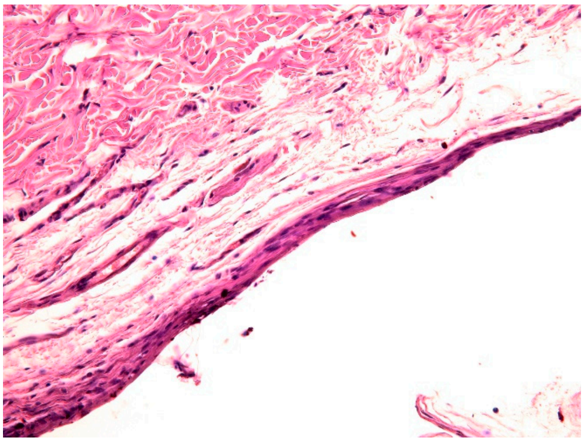

c)

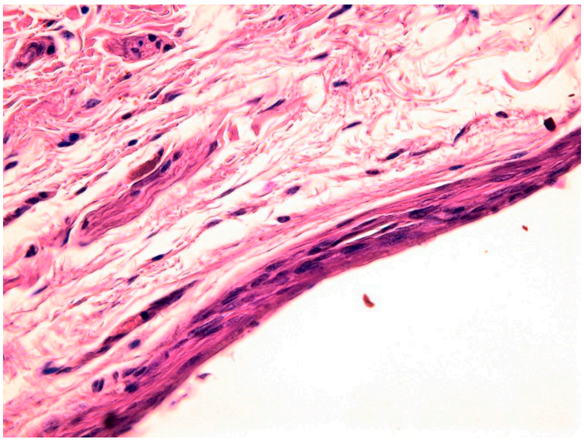

d)

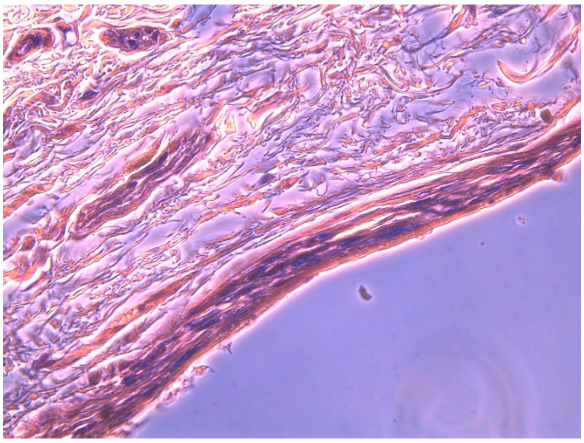

Figure S1. Autopsy of uncoated C-C composites at week 6 post-implantation

a) Standard light microscopy. Increase 50x. b) standard light microscopy. magnification 200x. c) standard light microscopy. 400x. d) phase-contract microscopy. 400x. H& E statining.

a)

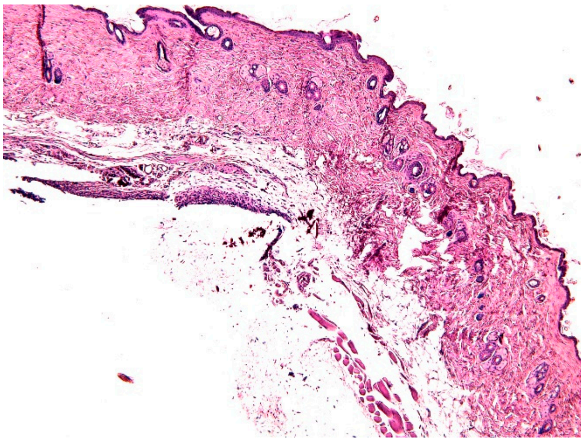

b)

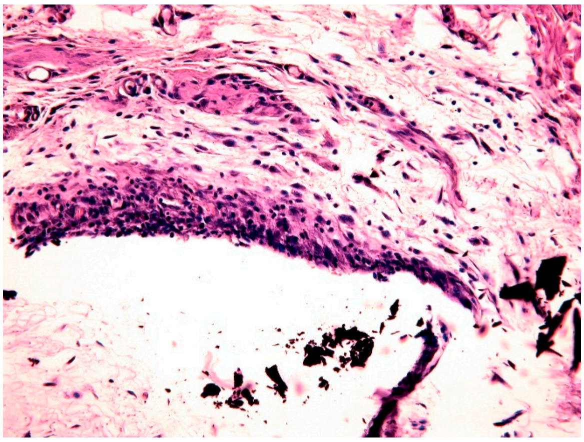

c)

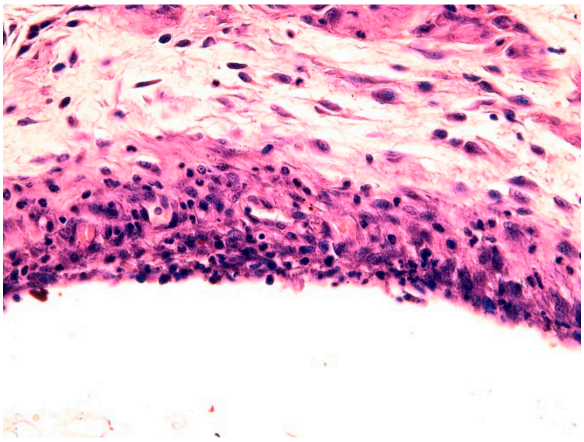

Figure S2. Autopsy of uncoated C-C composites at week 6 post-implantation with increased lymphocyte and macrophage infiltration

a) Standard light microscopy. Increase 50x. b) standard light microscopy. magnification 200x. c) standard light microscopy. 400x.

a)

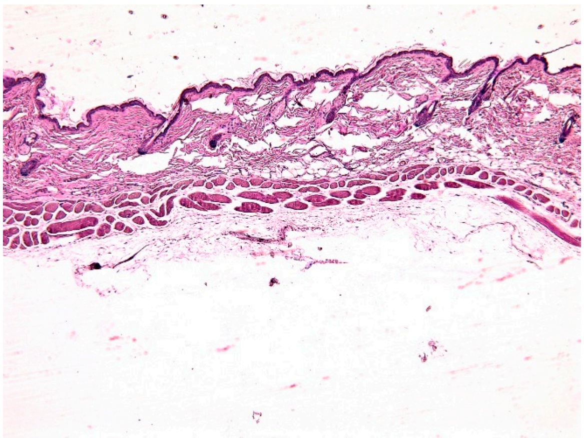

b)

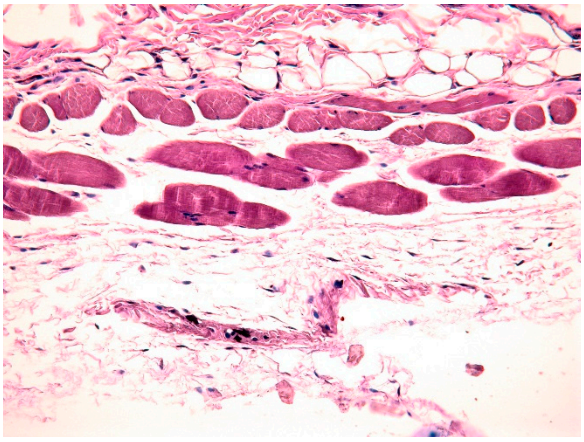

c)

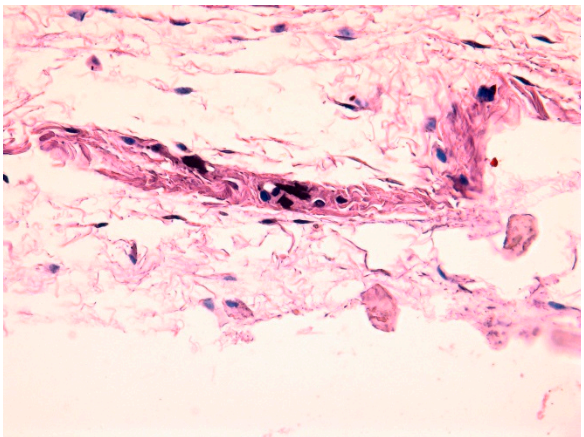

d)

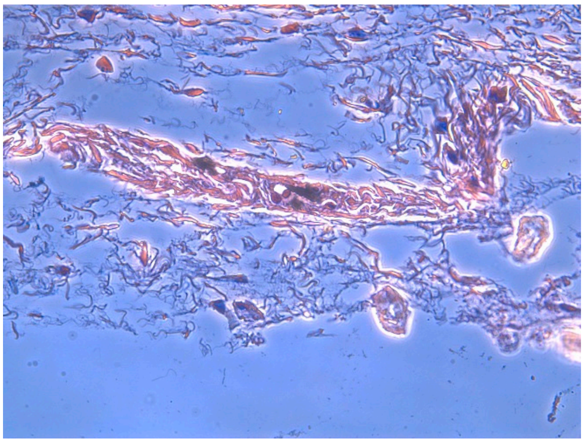

Figure S3. Aautopsy of uncoated C-C composites at week 12

a) Standard light microscopy. Increase 50x. b) standard light microscopy. magnification 200x. c) standard light microscopy. 400x. d) phase-contract microscopy. 400x. H& E statining

a)

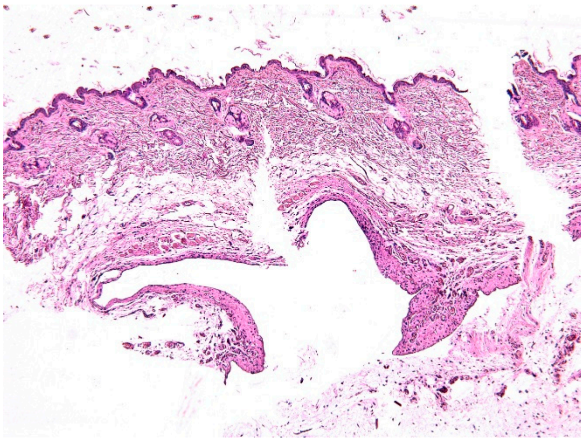

b)

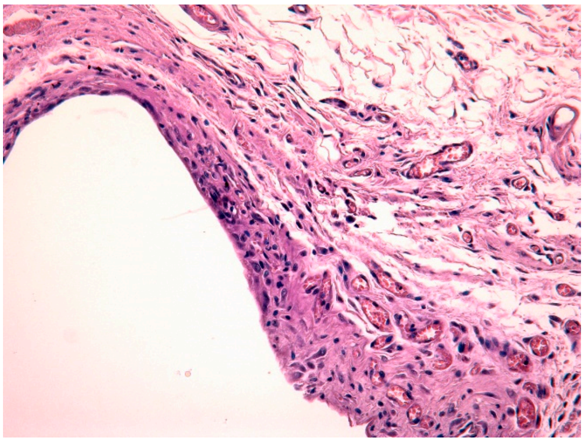

c)

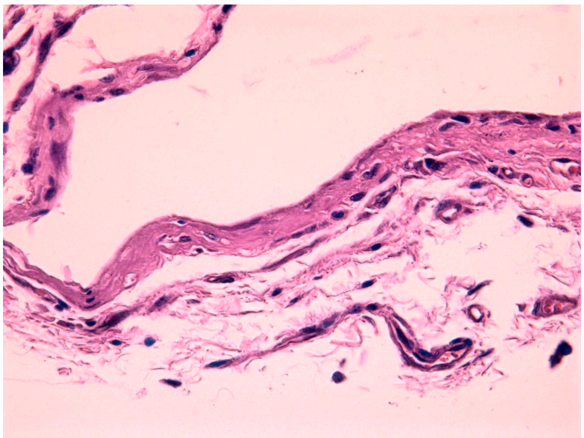

d)

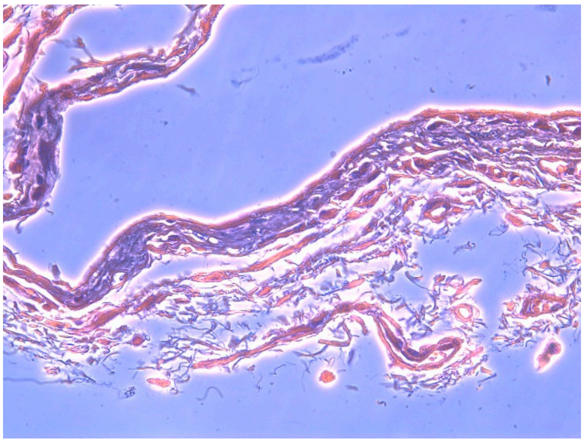

Figure S4. Autopsy of Ca-P coated (electrophoretic deposition) C-C composites at week 6 post-implantation

a) Standard light microscopy. Increase 50x. b) standard light microscopy. magnification 200x. c) standard light microscopy. 400x. d) phase-contract microscopy. 400x. H& E statining

a)

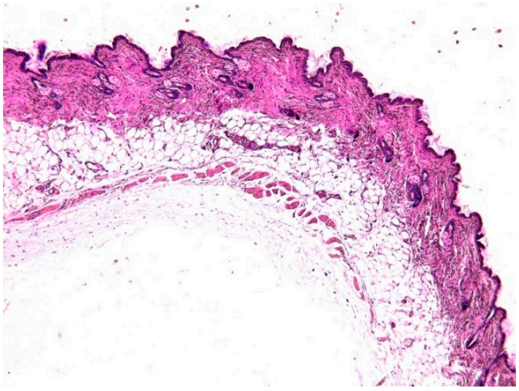

Figure S5. Autopsy of Ca-P coated (electrophoretic deposition) C-C composites at week 12 post-implantation

a) Standard light microscopy. Increase 50x. . H& E statining.

a)

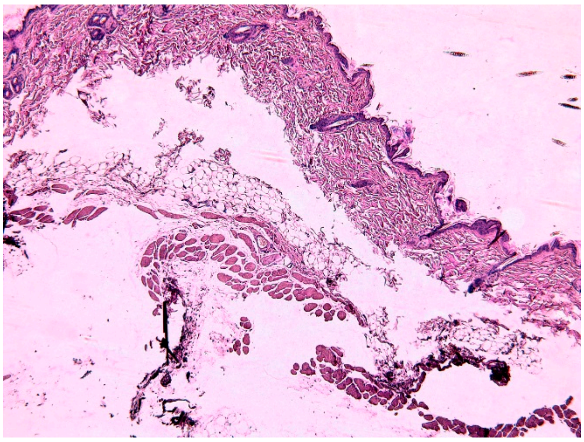

b)

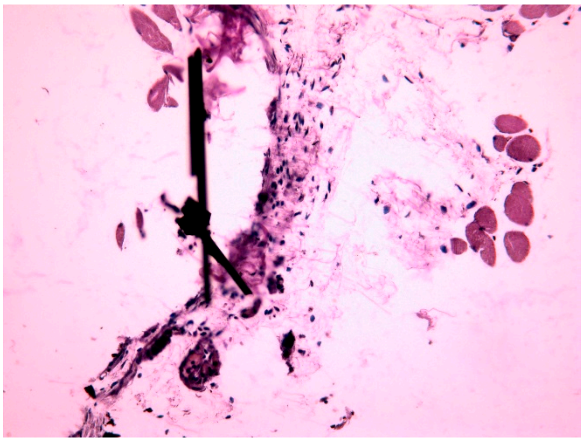

c)

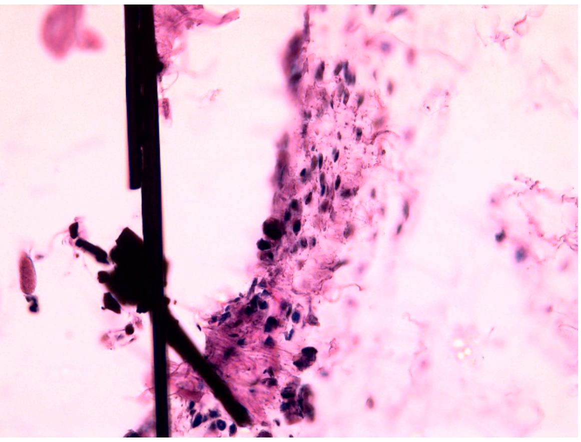

d)

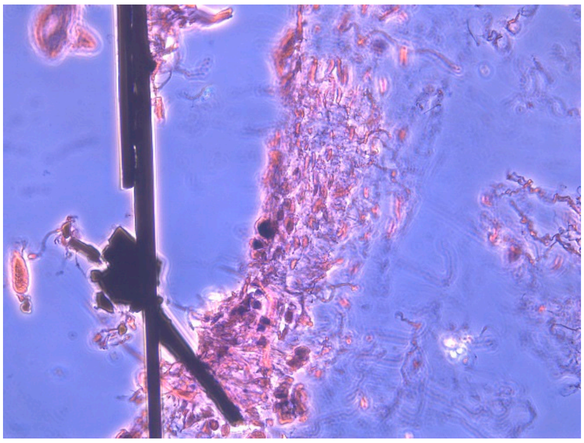

Fgure S6. Autopsy of Ca-P coated (detonation spraying) C-C composites at week 6 post-implantation

a) Standard light microscopy. Increase 50x. b) standard light microscopy. magnification 200x. c) standard light microscopy. 400x. d) phase-contract microscopy. 400x. H& E statining

a)

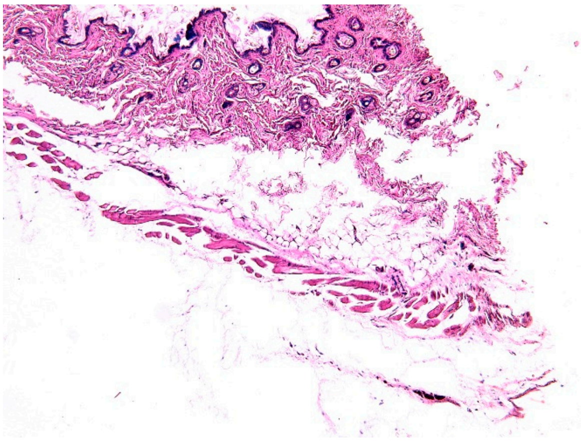

b)

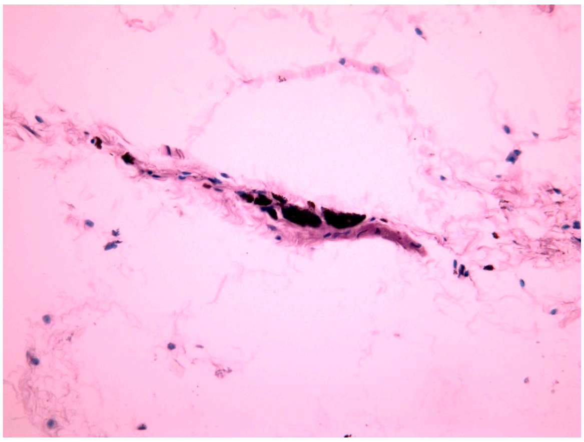

c)

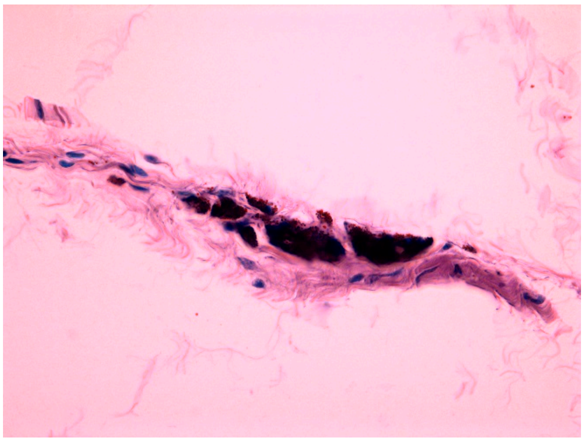

d)

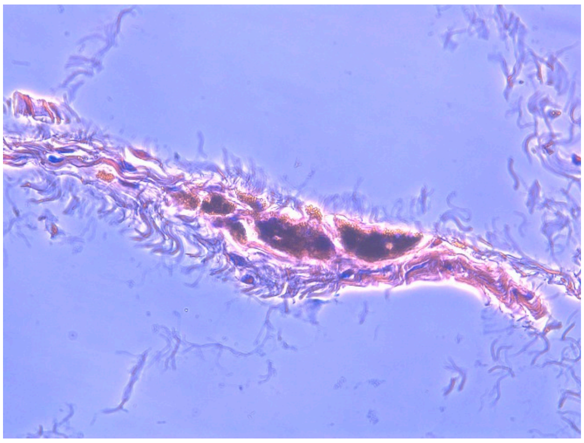

Figure S7. Autopsy of Ca-P coated (detonation spraying) C-C composites at week 12 post-implantation. *a) Standard light microscopy. Increase 50x. b) standard light microscopy. magnification 200x. c) standard light microscopy. 400x. d) phase-contract microscopy. 400x. H& E statining*
